# Innate Pathways of Immune Activation in Transplantation

**DOI:** 10.1155/2010/826240

**Published:** 2010-08-31

**Authors:** Todd V. Brennan, Keri E. Lunsford, Paul C. Kuo

**Affiliations:** ^1^Division of Transplantation, Department of Surgery, Duke University Medical Center, P.O. Box 3512, Durham, NC 27710, USA; ^2^Department of Surgery, Duke University Medical Center, P.O. Box 3512, Durham, NC 27710, USA

## Abstract

Studies of the immune mechanisms of allograft rejection have predominantly focused on the adaptive immune system that includes T cells and B cells. Recent investigations into the innate immune system, which recognizes foreign antigens through more evolutionarily primitive pathways, have demonstrated a critical role of the innate immune system in the regulation of the adaptive immune system. Innate immunity has been extensively studied in its role as the host's first-line defense against microbial pathogens; however, it is becoming increasingly recognized for its ability to also recognize host-derived molecules that result from tissue damage. The capacity of endogenous damage signals acting through the innate immune system to lower immune thresholds and promote immune recognition and rejection of transplant grafts is only beginning to be appreciated. An improved understanding of these pathways may reveal novel therapeutic targets to decrease graft alloreactivity and increase graft longevity.

## 1. Adaptive and Innate Immune Responses

Alloantigen-specific T cells and B cells cause acute cellular and humeral rejection through the recognition of graft antigen by highly evolved immune receptors. These receptors, T cell receptors and immunoglobulins, are capable of recognizing an immense variety of antigens due to their numerous encoding genes and due to the process of somatic rearrangement of their encoding DNA. The immense diversity of the cell receptors also predicates that for a novel antigen, only a limited pool of lymphocytes will have specificity towards that antigen. Consequently, in order to conduct an effective immune response, intense expansion of antigen-specific lymphocytes is required. Because this expansion may take several days, a more immediate defense system is also required to address microbial invasions that are capable of rapid progression. 

The innate immune system has come to the forefront of immunological research with the discovery of Toll-like receptors (TLRs) (reviewed in [1, 2]) along with the appreciation that the context in which the antigen is recognized is critical for promoting the immune response [3]. TLRs are pattern recognition receptors (PRRs) that are expressed on both nonlymphoid and lymphoid tissues, especially antigen- presenting cells such as dendritic cells and macrophages. Their ligation initiates intracellular signal transduction cascades that lead to NF-*κ*B activation and the upregulation of the adhesion molecules, costimulatory molecules and cytokines that are essential to immune activation [4, 5]. Characterization of the ligands and function of the various TLRs has revealed that the innate pathways are critical to the development of a robust adaptive immune response [1–3, 6–9].

Unlike the immensely variable antigen recognition epitopes of T cell receptors and antibodies, TLRs have a fixed genomic structure and are capable of binding a limited repertoire of ligands. Some of the resultant lack of variability is overcome by the presence of multiple receptor types; for example, there are currently 13 TLRs identified in mice and humans. Despite their limited antigen recognition capability, their conservation between evolutionarily distant species hints that they may bind molecules that are indispensible to microbes such that they cannot be mutated or ablated. The benefit of the TLR fixed receptor structure is that a large number of innate immune cells can recognize a pathogen and respond immediately.

## 2. Exogenous and Endogenous TLR Ligands

**Figure 1:**
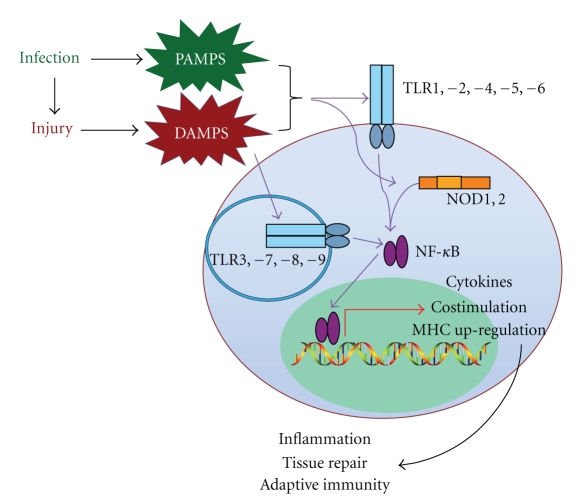
Infection and cell injury result in the production of PAMPs and DAMPs that promote the inflammatory response via TLRs located on the cell membrane and within endosomes. Cytoplasmic PAMPs activate similar pathways by binding to NOD1 and NOD2.

TLRs have been identified with affinities for molecules associated with infection and tissue injury. However, their ability to recognize pathogen-associated molecular patterns (PAMPs) is best described. Some TLRs (TLR1, −2, −4, −5 and −6) are located on the outer cell membrane and recognize microbial molecules derived from bacteria, fungus, and parasites (reviewed by Akira et al., 2006 [1]) (Figure 1). For example, TLR2 recognizes bacterial peptidoglycan, fungal phospholipomannan, and Trypanosomal tGPI-mutin and TLR4 recognizes bacterial lipopolysaccharide (LPS), fungal mannan, and Trypanosomal Glycoinositol phospholipids. Other TLRs (TLR3, −7, −8 and, −9) are located within the in the endosomal/lysosomal compartment and bind bacteria- and virus-derived nucleic acids. For example, TLR3 binds viral double-stranded RNA, TLR7 and TLR8 bind viral single-stranded RNA, and TLR9 binds bacterial and viral double-stranded DNA.

TLRs share homology with the Type I transmembrane Toll receptor discovered in the fruit fly (*Drosophila melanogaster*) initially identified for its role in controlling dorsal-ventral polarity during embryogenesis [10]. It was later discovered that Toll also induces production of antimicrobial peptides in response to fungal infection in adult fruit flies [11]. Considering the role of the Toll receptor in development as well as primitive innate immunity, it is not surprising that TLRs have endogenous ligands in addition to microbial ligands. Endogenous TLR ligands arising from tissue damage are termed damage-associated molecular patterns or “DAMPs”, and they are becoming increasingly recognized for their role in immune regulation (Figure 1) [12–14]. 

More than 20 DAMPs have been described as stimulants for TLRs [15]. Examples include heat-shock protein 60 (Hsp60), Hsp70, surfactant protein A, *β*-defensin 2 high-mobility-group box 1 protein (HMGB1), and extracellular matrix molecules such as hyaluronan, fibronectin, and and heparan sulfate [16, 17]. Some controversy exists with regards to potential contamination of DAMPs with PAMPs (e.g., LPS) resulting in false positive results of TLR stimulation [18–20]. This is especially relevant to protein stimulators that have been synthesized from bacterial recombinant technology. Nonetheless, there is accumulating evidence for the role of DAMPs in shaping the overall immune response especially when derived from stressed, injured, or necrotic cells [21, 22].

While multiple TLRs exist, they share common intracellular signaling pathways [4]. These include myeloid differentiation primary response protein (MyD88), through which all TLRs signal with the exception of TLR3, which utilizes TRIF (Toll/IL-1R domain–containing adaptor inducing IFN-*α*) [5]. Signal transduction pathways though both MyD88 and TRIF have been described for TLR4 [5]. These pathways converge with the activation of NF*κ*B, which results in the transcription of multiple immune stimulatory genes involved in immune cell development, maturation and, cytokine production and proliferation [23, 24].

## 3. TLRs in Transplantation

Several recent studies have highlighted the importance of the innate immune system in allograft rejection in mouse and human transplantation. In a minor antigen mismatch model of graft rejection using HY-mismatched skin grafts, MyD88 knockout recipients showed transplant survival >100 days whereas wild-type recipients rejected skin allografts at a median of 16 days [25]. Interestingly, selective deletion of TLR2 alone (TLR2 knockout hosts) only slightly prolonged skin allograft survival, and deletion of TLR4 (TLR4 knockout hosts) did not prolong skin allograft survival [25]. These results imply that other MyD88-dependent pathways contribute to alloimmunity (e.g., IL-1) or that significant redundancy in the signaling pathways exists. In contrast, MyD88 deficiency does not prolong survival of fully mismatched allogeneic skin and heart transplants [26]. However, in both the minor and major mismatch experiments, MyD88 deficiency leads to a defect in Th1- dependent alloimmunity [25, 26]. This suggests that the MyD88 pathways skew the immune response towards Th1- type immunity and is sufficient to mediate allograft rejection when only minor antigen mismatches are present. However, this effect is outweighed by the stronger immunologic stimuli of a full antigen mismatch. 

Polymorphisms of TLR4 at Asp299Gly and Thr399Ile cause endotoxin hyporesponsiveness, and these mutations are relatively common in the human population [27]. Kidney transplant recipients who carry these TLR4 polymorphisms have been noted to have fewer episodes of acute rejection [28]. This same TLR4 polymorphisms decrease the incidence of acute allograft rejection when present in lung transplant donors, but not recipients [29]. A trend toward reduced acute graft-versus- host disease following bone marrow transplantation is noted when either the bone marrow donor or recipient carries these polymorphisms, but the effect is greater when the recipient carries the TLR4 mutation [30]. Interestingly, hepatitis C-infected liver transplant recipients with the TLR4 Asp299Gly mutation are found to have significantly worse long-term graft outcomes than recipients lacking this mutation [31]. Overall, these studies provide clinical substantiation of the experimentally observed importance of TLR4 in graft rejection.

## 4. Innate Immune Activation in Transplantation-Tissue Injury

**Figure 2:**
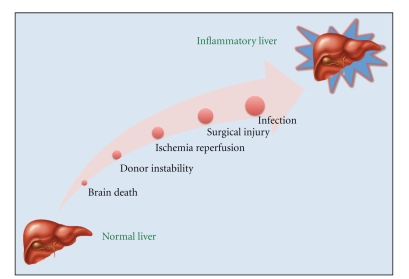
Liver injury during transplantation can convert an immunologically quiescent organ to an inflammatory organ that promotes acute rejection.

Tissue injury during the pre- and posttransplant periods may result from a multitude of mechanisms, and these factors contribute to end-organ damage and affect allograft survival (Figure 2). Immune activation in the donor organ during the pretransplant period begins with brain death and the neuropathology associated with brainstem herniation. As the medulla becomes ischemic, vagal activity ceases, resulting in massive sympathetic outflow and high levels of catecholamines. In addition to affecting cardiac function by stimulating tachycardia, hypertension, and dysrhythmia, catecholamine release results in peripheral vasoconstriction that can contribute to end-organ ischemia. Following the resolution of the catecholamine storm, the sympathetic tone is lost, resulting in vasodilatation and reperfusion injury to tissue followed by hypotension that can again cause organ hypoperfusion. Brain death also results in an outpouring of inflammatory cytokines [32], including IL-6 that has been shown to correlate with worse recipient outcomes in the setting of kidney transplantation [33]. 

In addition to the release of a multitude of cytokines, acute brain injury has also been shown to up-regulate endogenous innate immune activators Hsp70 [34] and HMGB1 [35], as well as to cause the release of fibrinogen fragments [36]. Interestingly, while inflammation derived from acute brain injury has been shown to be dependent on MyD88, it has been shown to be independent of TLR2 and TLR4, the receptors identified for the majority of DAMPs [37]. Potentially, alternative pathways of MyD88-mediated TLR signaling act to transduce these inflammatory signals. 

The effect of adverse proinflammatory and neurophysiologic events on the donor organ quality that arise from brain death has led to the initiation of several studies investigating preprocurement donor cytoprotective therapies. For example, dopamine pretreatment has been observed to protect rat kidney allografts from cold ischemic injury [38, 39], potentially by augmenting the expression of the heat-shock protein, heme-oxygenase-1 [40, 41]. Recently, in a randomized controlled study of human kidney transplantation, donor pretreatment with dopamine significantly improved early graft function [42]. Donor pretreatment with intraperitoneal glutamine in the rat kidney transplant model diminishes early structural damage due to prolonged preservation reperfusion injury [43]. Preconditioning with oral vitamin E has also been noted to improve post-ischemic recovery of systolic function following rat cardiac transplantation [44]. These experimental therapies suggest future potential to improve the viability of deceased donor organs.

Necrotic cell death that can result from cold ischemia, ischemia-reperfusion injury, and surgical trauma has been shown to elicit a strong inflammatory response [45]. For example, necrotic or damaged cells release HMGB1, a chromatin-binding protein that can act as an endogenous activator of TLR4 and cell signaling mediated by MyD88 when released extracellularly [46, 47]. HMGB1 can be secreted by activated monocytes and macrophages and is passively released during cellular necrosis. HMGB1 is a potent promoter of inflammation that results in the release of cytokines and chemokines that promote inflammatory tissue damage [47, 48]. 

Surgical trauma to the donor organ incurred during procurement and to the donor and recipient during the transplantation procedure can release DAMPs and activate the immune system. However, probably the most significant contributor to either organ or end-organ injury during transplantation is the ischemic injury caused by cold storage followed by the warm reperfusion at the time of engraftment. The association between rejection and increased duration of cold ischemia has been well established [49]. Modulation of reperfusion damage may result in improved allograft function following transplantation. For example, fingolimod (FTY720), a sphingosine-1-phosphate receptor agonist that interferes with lymphocyte trafficking, provides tubular epithelial protection in the presence of severe preservation-reperfusion injury in a rat kidney transplant model [50].

Reperfusion of ischemic organs results in activation of inflammatory pathways and complement cascades that increase graft immunogenicity [51–53]. Murine models of ischemia-reperfusion injury provide evidence for the role of TLRs in mediating reperfusion injury. In mouse models of kidney reperfusion injury, both TLR2 and TLR4 expressions are increased amongst renal epithelial cells [54]. Similarly, in models of myocardial ischemia-reperfusion injury, mice deficient in TLR4 develop smaller areas of myocardial infarction when compared to injury in wild-type mice [55]. TLR4 has also been shown to be a key factor in liver ischemia-reperfusion injury [56].

## 5. Innate Immune Activation in Transplantation-Infection

Following transplantation, the liver remains susceptible to additional sources of innate immune activation from infection. Microorganisms, either from bacteria translocation across injured bile duct epithelium or from post-operative infections in the form of bilomas, abscesses, wound infections, and viral infections, may initiate inflammatory cascades that adversely affect the allograft survival. Inflammatory responses due to systemic viral infections such as cytomegalovirus (CMV) [57], herpes viruses [58], adenovirus [59], and polyomaviruses [60]; moreover viral infections within the transplanted organ such as hepatitis B [61] and hepatitis C liver infections [62], adenovirus heart infection [63], and CMV graft infections [58, 64, 65] have been associated with adverse clinical outcomes. In experimental models, systemic viral infections are also known to result in allograft rejection and disruption of immunoregulation [66–70].

The activation of innate immune pathways is likely important in directing the initial activation of the allograft rejection response, but they also may disrupt established immune tolerance. Stimulation of TLR receptors with LPS or CpG DNA has been shown to abrogate transplant tolerogenic regimens in both skin and heart transplant models [71, 72]. While the mechanism of the break in tolerance is not well understood, it may be caused by the failure of graft-reactive CD8^+^  T cells to undergo apoptosis [71], the blockade of Treg function [73], or the accumulation of Tregs at the graft site [71].

## 6. Future Area of Investigation

Although multiple pathways by which DAMPs may activate innate immune responses have been discovered, additional pathways await discovery. Different routes of cell death may release divergent signals to the immune system. For example, programmed cell death through apoptosis may release different immune mediators than cells that died from stress or injury. Evaluation of lysates of otherwise healthy cells made necrotic by Dounce-lysis or freeze thaw cycles, demonstrates that they contain factors that are able to induce NF-*κ*B via TLR2 present on fibroblasts and macrophages [74]. The activating agent must be present in healthy cells at the time of lysis and does not require de-novo synthesis. In contrast, cells made apoptotic through irradiation did not induce NF-*κ*B  [74]. This demonstrates that not all dead cells are equally stimulatory and that genetic programs may exist to sequester DAMPs when programmed cell death occurs. 

Evaluation of a different cell fraction may also illuminate a larger variety of DAMPs. For example, mitochondria are intracellular organelles with bacterial origins and have recently been discovered to harbor DAMPs. Cells made necrotic through freeze-thaw cycling release mitochondrial *N*-formyl peptides that stimulate IL-8 production by monocytes [75]. Similar to bacteria, mitochondrial DNA is rich in CpG dinucleotides which are the ligand for TLR9 in monocytes [1]. In addition, DAMPs can represent organic products of metabolism. For example, uric acid has been recently shown to elicit an acute inflammatory response to sterile cell death in mice [76].

New research has implicated many DAMPs to be molecules that increase the efficacy of PAMPs rather than being true TLR ligands themselves [15]. Further research into the precise mechanism of DAMP-TLR binding needs to be done to confirm which DAMPs are ligands and which only facilitate the binding of true ligands. It also remains unclear whether receptor competition exists when multiple DAMPs are present simultaneously, as would be expected at sites of injury. Similarly, there may exist competition between DAMPs and PAMPs for TLRs. Alternatively, DAMP- and PAMP-mediated cell signaling may synergize. 

Additional pathways of molecular signaling that contribute to the propagation of innate immune signals need to be further investigated. For example, several microRNAs (miRNAs) have been found to regulate the innate response to pathogens [77]. miRNAs are a recently described family of small, noncoding RNAs that regulate gene expression by interfering with protein translation and by targeting messenger RNA for degradation. Already there is evidence that miRNA-146 serves as a negative feedback inhibitor of TLR signal transduction [78] and that miRNA-125b and miRNA-155 regulate the stimulation of TNF-*α* production by the innate immune system [79]. 

The role of non-TLR innate receptor families in the regulation of the immune response is also just beginning to be uncovered. For example, the NOD-LRR and CARD-helicase proteins, which comprise a huge family of receptors involved in pathogen recognition [80, 81], have only recently been defined. Unlike TLRs, which are imbedded in cell surface or lysosomal-endosomal membranes, these receptors are cytosolic and recognize pathogen-associated molecules within the cytosol. Like TLRs they can produce an inflammatory response driven by NF-*κ*B thus, resulting in immune activation. One member of the NLR family, NLRP3 (NLR family, pyrin domain containing 3) has been found to be involved with the sterile inflammatory response caused by necrotic cells [82] and in the stimulation of IL-1*β* secretion triggered by cholesterol crystals [83].

We need to further our understanding of the innate immune pathways that contribute to the alloimmune response leading to acute, as well as chronic, graft rejection. These studies need to look at the contributions of both exogenous and endogenous innate immune stimulants and how these two sources of ligands may function in synergistic activation pathways. Also, some ligands may function as competitive inhibitors, and their role in immune suppression could provide a novel route of immunosuppression. Finally, targeting the innate pathways can be instituted at multiple timepoints in the transplant setting: in the donor beginning with brain death, during procurement, cold storage, reperfusion, immediately postoperatively, or in the late postoperative period in the setting of infection or chronic rejection. How and when to address these pathways has yet to be determined.

## 7. Summary

As our understanding of the immune systems grows, the mechanisms by which effective allograft rejection responses are initiated become increasingly complex. The role of allogeneic T cells and B cells in precipitating rejection has been well established; however, more recent investigations have highlighted the way in which innate immune responses may skew or direct adaptive immunity. The chief among these pathways appears to be the TLRs. Although evolutionarily primitive, these receptors appear to propagate innate immune activation and to facilitate activation of adaptive immunity in ways that are only presently being elucidated. In the case of allograft immunity, initiation of innate immune signals through DAMPs and PAMPs can activate potent immune stimulatory pathways that increase allograft vulnerability to the host immune system. Strategies for successful modulation of these signals will likely improve allograft outcomes and allow for the minimization of systemic immunosuppressive therapies.
